# Trafficked Proteins—Druggable in *Plasmodium falciparum*?

**DOI:** 10.1155/2013/435981

**Published:** 2013-04-28

**Authors:** Jasmin Lindner, Kamila Anna Meissner, Isolmar Schettert, Carsten Wrenger

**Affiliations:** ^1^Unit for Drug Discovery, Department of Parasitology, Institute of Biomedical Science, University of São Paulo, Avenida Prof. Lineu Prestes 1374, 05508-000 São Paulo, SP, Brazil; ^2^Laboratory of Genetics and Molecular Cardiology, Heart Institute InCor, Avenida Dr. Eneas de Carvalho Aguiar 44, 05403-000 São Paulo, SP, Brazil

## Abstract

Malaria is an infectious disease that results in serious health problems in the countries in which it is endemic. Annually this parasitic disease leads to more than half a million deaths; most of these are children in Africa. An effective vaccine is not available, and the treatment of the disease is solely dependent on chemotherapy. However, drug resistance is spreading, and the identification of new drug targets as well as the development of new antimalarials is urgently required. Attention has been drawn to a variety of essential plasmodial proteins, which are targeted to intra- or extracellular destinations, such as the digestive vacuole, the apicoplast, or into the host cell. Interfering with the action or the transport of these proteins will impede proliferation of the parasite. In this mini review, we will shed light on the present discovery of chemotherapeutics and potential drug targets involved in protein trafficking processes in the malaria parasite.

## 1. Introduction

Malaria is a disease of global importance resulting in morbidity and mortality worldwide [1, 2]. Although, since 2000 a reduction in the mortality rate of about 25% has been globally observed, nearly half of the world's population is still living at risk of infection [1, 3] leading to more than 0.5 million deaths annually [4]. 

Five species of the genus *Plasmodium* are known to infect humans; however, the most severe form of malaria, Malaria tropica, is caused by* Plasmodium falciparum *[5, 6].

Due to the spreading drug resistance against the currently used chemotherapeutics as well as environmental changes, the treatment of the disease is becoming more difficult. Resistance to therapeutics such as chloroquine and sulfadoxine-pyrimethamine is already widely distributed [28–31]. Furthermore resistance to Malarone, a combination of atovaquone and proguanil, became also apparent [32]. Currently only ACTs (artemisin-based combination therapies) are highly effective against malaria [33]. However, resistances to artemisinin and its derivatives have already been confirmed at the border of Cambodia-Thailand [34–36], and therefore the discovery of novel therapeutic agents against malaria is urgently required.

Proliferation of *P. falciparum* within the asexual blood stage depends on haemoglobin digestion in the food vacuole [37]. This degradation pathway was already taken into consideration as a new drug target by focussing on the proteases plasmepsin I–IV [25]. However, since haemoglobin catabolism occurs in the food vacuole, not only inhibition of the catalytic properties of the digestive enzymes is of interest but also the mode of protein trafficking. The latter was also considered to be druggable for proteins directed to other parasite-specific organelles such as the chloroplast-like organelle, the apicoplast, where several essential metabolic processes take place. Additionally to the specific intraparasitic trafficking the malaria pathogen secretes also a variety of proteins to the erythrocytic host cell surface for nutrient transport processes as well as for cytoadherence mediated by proteins for example of the *Plasmodium falciparum* erythrocyte membrane protein (*Pf*EMP)-family. Interestingly, the number of exported proteins is—in the case of *P. falciparum*—about 5–10 times larger compared to other malaria parasites [38]. Therefore, protein trafficking in *P. falciparum* represents an attractive drug target; however, detailed information of its mode of action is required to interfere with this mechanism. The aim of this mini review is to summarise the current knowledge of novel promising drug targets, which are involved in protein trafficking processes in *P. falciparum*.

## 2. Protein Trafficking in *P. falciparum*

Like other eukaryotic cells,* P. falciparum* possesses intracellular compartments such as a nucleus, endoplasmic reticulum (ER) and a mitochondrion [39]. However, the pathogen also possesses an apicoplast.

Although the apicoplast has its own circular genome encoding for about 30 proteins, the majority of the apicoplast located proteins are nuclear encoded in *P. falciparum *[40, 41]. Around 500 proteins are predicted to be trafficked to this organelle, like the enzymes of the isoprenoid fatty acid and iron-sulfur-cluster biosynthesis pathways [42, 43]. These proteins possess target information for the transport to the apicoplast, which is encoded at the N-terminal region [44]. First, the signal peptide, a hydrophobic N-terminal sequence, directs the proteins to the ER lumen [45–47], where subsequently a signal peptidase cleaves off the signal peptide [48]. Within the N-terminal region the signal peptide is followed by a second domain, the plant-like plastid transit peptide, which contains the information for trafficking the protein to the apicoplast [49]. 

The vesicular transport to the food vacuole is not yet completely understood. While some proteins like the chloroquine resistance transporter (*Pf*CRT), which modulates resistance to drugs like chloroquine, are transported directly via the classical ER and Golgi secretory pathway to the food vacuole [50–53], other proteins such as plasmepsin I, plasmepsin II or dipeptidyl aminopeptidase 1 (DPAP1), and the secreted acid phosphatase (SAP) are suggested to be targeted via the parasite plasma membrane (PPM) [20, 54] or the parasitophorous vacuole to the food vacuole [55]. The occurrence of different trafficking routes is also emphasised by the sensitivity to Brefeldin A, an inhibitor of the Golgi apparatus [56, 57]. Whereas the routing mechanisms of some proteins are affected by Brefeldin A, other proteins can still be found in the food vacuole after treatment with the inhibitor [58] leading to the suggestion of at least two different transport pathways, ER/Golgi dependent and independent trafficking routes.

Since transport to the food vacuole differs, consequently the signal sequences are very divergent. While for plasmepsin IV the first 70 amino acid residues are necessary for proper translocation, a mislocalisation of plasmepsin II occurs when different positions in the first 120 amino acid residues are deleted or mutated [59, 60]. Moreover, signal peptides of other hemoglobinases like DPAP1 are not yet analysed in detail. 

Additionally to the intraparasitic protein trafficking, *P. falciparum *secretes proteins to extracellular compartments like the parasitophorous vacuole, the Maurer's clefts, and into the host cell cytosol. Therefore, proteins have to enter the classical secretory pathway by a conventional signal peptide. Since the signal peptide is unable to transport a protein through the parasitophorous vacuole membrane a second signal sequence of around 25–30 amino acid residues after the ER signal (RxLxE/Q/D) called the *Plasmodium* export element (PEXEL), or vacuolar translocation signal (VTS), is necessary [61, 62]. These transport signals are conserved in *Plasmodium* spp. [62]. As about 8% of the parasitic proteome contains this targeting sequence, it is expected that most them have an important role in remodelling the host cell [61–63]. A knockout screen of 85 out of the predicted 200–300 exported proteins suggests that about 25% are essential in the blood stage of *P. falciparum *[38]. After cleaving off the PEXEL motif via the aspartic protease plasmepsin V, the proteins are transported to the parasitophorous vacuole [64]. Within the parasitophorous vacuole membrane the ATP-driven *Plasmodium* translocon of exported proteins (PTEX)-complex transports the released proteins across this barrier [65, 66].

However, *P. falciparum* possesses also a PEXEL-independent trafficking pathway consisting of the PEXEL-negative exported proteins (PNEPs) [67]. So far no consensus sequence for the export of PNEPs has precisely been identified, therefore it is hard to predict how many unidentified PNEPs are encoded by *P. falciparum*.

## 3. Proteins of Interest within the Apicoplast

One of the most interesting compartments in *P. falciparum* is the four membrane-bound, nonphotosynthetic organelle (the apicoplast), which has been proposed to be derived from secondary endosymbiosis. Briefly, while the uptake of a prokaryote by an eukaryote is termed primary endosymbiosis resulting in a plastid surrounded by two membranes, secondary endosymbiosis, the engulfment of an eukaryote possessing a primary endosymbiont, is leading to a four membrane-bound plastid [68]. 

As already outlined above, most of the apicoplast proteins are encoded in the nuclear genome and are trafficked to the organelle [12, 69–71]. Import into the plasmodial plastid is—in contrast to plants—a two-step process requiring a signal peptide and subsequently a transit peptide [43, 49]. Whereas the signal peptide mediates entry into the endomembrane system, the transit peptide directs the protein into the apicoplast [49, 72]. Plasmodial transit peptides are highly enriched in lysine and asparagine, and acidic residues are depleted suggesting that these characteristics are essential for plastid targeting in *P. falciparum*, as has been observed in mitochondrial transit peptides [73, 74]. As apicoplast transit peptides do not possess a highly conserved sequence motif [75] (they are varying widely in their length and their composition of positively charged amino acid residues near the N-terminus [42, 76]), it remains unclear how apicoplast proteins are distinguished from proteins which are intended to be translocated to other compartments. The translocation across the apicoplast periplastid membrane (second membrane from the cytosol direction) is assumed to be initiated by a novel ER-associated degradation (ERAD) complex [77]. The luminal chaperone binding protein (BiP) and protein disulphide isomerase (PDI) differentiate misfolded proteins from folding intermediates and correctly folded proteins [78–80]. Subsequently, misfolded proteins which are recognised by ERAD are redirected to the cytosol across the ER membrane through a channel which consists of either Sec61 [81] or Der1 [82, 83].

Another factor suggested to play a role in protein targeting to plant plastids is the binding of HSP70 chaperones to plastid transit peptides [84, 85]. A specific HSP70 binding pattern is present, and putative binding sites are abundant in apicoplast transit peptides. Therefore, an important role of HSP70 in apicoplast targeting has been suggested [85]. 15-Deoxyspergualin (DSG) is an immunosuppressant with antimalarial activity acting for example on HSP70 molecules within the malaria parasite [7, 8] (Table 1). The mode of action is to date unknown, but it is assumed that DSG inhibits chaperone-transit peptide interactions, and thereby preventing apicoplast targeting [9]. Another class of drugs interfering with apicoplast targeting is the group of the tetracyclines such as doxycycline (Table 1). Tetracyclines are broad-spectrum antibiotics working as protein synthesis inhibitors by binding to microbial ribosomes and blocking the translation process. The inhibition by tetracycline and related drugs occurs probably indirectly by inhibiting the translation of apicoplast-encoded genes such as the ClpC gene which is required for protein import. Further drugs beside tetracycline acting via this mechanism are clindamycin and azithromycin [86].

Currently no clinical resistance to doxycycline has been reported [10]. Whereas doxycycline presumably targets the translation within the apicoplast, rifampicin has been proposed to attack transcription; both drugs result in delayed death (Table 1). Unfortunately the versatile use of the latter supports the development of drug resistance against these antibiotics [87]. The synthesis of isoprenoid precursors is an essential metabolic function of the apicoplast. In *P. falciparum, *isoprenoids are *de novo* generated via the DOXP (1-deoxy-D-xylulose-5-phosphate) pathway. As the canonical mevalonate pathway is apparently absent in *P. falciparum,* the apicoplast represents the only place within the parasite where isoprenoid precursor synthesis occurs [88, 89]. This biosynthetic pathway consists of seven enzymes, and all are apparently encoded in the nucleus and their deriving proteins are targeted to the apicoplast [88]. The inhibition of the DOXP reductoisomerase/IspC by fosmidomycin results in death of the *P. falciparum* intraerythrocytic stages which underlines the important role of this pathway for the malaria parasite [12] (Table 1). Moreover, the inhibition of another enzyme of the DOXP pathway, IspF, also leads to the death of the parasite's intraerythrocytic stages [90]. Therefore the apicoplast isoprenoid pathway represents a promising target for the development of novel antimalarials. However, the discovery and validation of the organelle pathways are hindered either by limitations to apply reverse genetics of essential genes or to characterize plasmodial proteins *in vitro* or to purify the organelle [91–94]. 

## 4. Interfering with Proteins Trafficked to the Food Vacuole

The digestion of haemoglobin is carried out by the malaria parasite within the food vacuole, an organelle that has an acidic pH [95, 96]. Haemoglobin is taken up via endocytosis and is suggested to be subsequently transported by lysosomal vesicles to the digestive vacuole [95, 97]. Haemoglobin degradation is substantially carried out by four different protease families. The initial degradation of the haemoglobin into globin fragments is mediated by aspartic proteases, such as the plasmepsins I,II,IV, and the histoaspartic protease (HAP) [20, 98–100]. In the next step, the cysteine proteases falcipain 2, 2b, and 3 [101, 102] digest these globin fragments into peptides which are subsequently degraded into smaller peptides by the metalloprotease falcilysin [103]. These polypeptides are cleaved into dipeptides by a dipeptidyl aminopeptidase and finally aminopeptidases convert the dipeptides into amino acids [104]. The proliferation of the parasite is repressed by protease inhibitors that block the haemoglobin catabolism. Therefore, these endo- and exopeptidases catalyzing this process are attractive targets for the development of novel antimalarial drugs [97, 105–107]. Recent analysis identified the fungal metabolite bestatin as an inhibitor of neutral aminopeptidases such as the M1 aminopeptidase (*Pf*A-M1) [18] and the aminopeptidase P (*Pf*APP) [95, 96]. Furthermore, it has been reported that this inhibitor, a natural Phe-Leu dipeptide analogue derived from the fungus *Streptomyces olivoretticuli*, prevents growth of the parasite *in vitro* and *in vivo*. [15–17]. Two phosphinate dipeptide analogues (compound **4** and **5**) were also effective against *P. falciparum* in cell culture [17] (Table 1). Both compounds inhibit the M17-family leucine aminopeptidase (*Pf*LAP) with IC_50_ values of 20−40 and 12−23 *μ*M, respectively (Table 1) [17]. Additionally, *in vivo* studies using *P. chabaudi* resulted in an about 90% reduction of parasitaemia in mice treated with compound **4** [17]. These results clearly confirm that neutral aminopeptidases are serving as promising new antimalarial drug targets [17].

In the food vacuole, haemoglobin degradation is initiated by the aspartate proteases, plasmepsin I-IV (III has been renamed to HAP), which can be inhibited by pepstatin A at the picomolar range [108]. However, at the cellular level, this molecule is only a weak inhibitor. Recently, it has been shown that knock outs of plasmepsins result in delayed growth but is not leading—even in the presence of specific protease inhibitors—to the death of the parasite [109, 110]. Currently the aim of a new initiative between the Johns Hopkins University and Kyoto Pharmaceutical University is focussing on designing “adaptive inhibitors” (KNI) which were able to inhibit all four digestive vacuole plasmepsins simultaneously (Table 1). The function of these peptidomimetic inhibitors is based on the strongest and most specific interactions of the inhibitor against the conserved regions of the binding site and at the same time maintaining flexibility of the inhibitor allowing docking to the less conserved regions [22–24]. KNI-10006 for example is very potent against plasmepsin II but has only poor influence on parasite growth. In contrast, compound KNI-10283, which does not contain a basic amine, has been tested for *in vivo* efficacy in the *P. berghei*-infected mouse model revealing an expanded increase in lifespan of mice of up to 170% [25, 111, 112]. These results support the development of broad-spectrum plasmepsin inhibitors as antimalarial agents. Another group of plasmepsin inhibitors are the nonpeptidomimetic inhibitors containing a number of different structural classes such as N-alkoxyamides, guanidines, amides, ureas, thioureas, hydrazides, and amino alcohols [112–117]. Whereas many of them are only poor inhibitors, such as halofantrine, some of them including ACT-056822, which extended the survival time of mice of about 160% in the *P. berghei* mouse model, seem to be promising plasmepsin inhibitors (Table 1).

## 5. Inhibition of the Export System

The export of proteins which are located at the surface of the host cell like *Pf*EMP1 is responsible for the virulence of *P. falciparum. Pf*EMP1 is involved in the knob assembly and is therefore important for mediating cytoadherence of the infected erythrocyte to the endothelial cell of the blood vessels by binding on DC36, ICAM-1, thrombospondin, chondroitin sulfate, and other surface molecules [118]. The inhibition of the export machinery seems to be an auspicious new druggable pathway. As already outlined above, a variety of these exported proteins are trafficked via the PEXEL motif to the surface of the host cell [38]. Indeed, the proteolytic enzyme plasmepsin V seems to be a promising drug target candidate, as it is conserved in *Plasmodium* spp. and is absent from higher eukaryotes. The fundamental role of this protease for the malaria parasite has been verified by the attempt to disrupt a plasmepsin V orthologous gene (*Pb*PMV) in the rodent malaria parasite *P. berghei *[64]. Plasmepsin V is localised in the ER and cleaves the PEXEL motif between position 3 and 4 of the PEXEL sequence [119]. This protease is therefore responsible for proper processing of PEXEL mediated transport processes to the host cell. Due to the different function of plasmepsin V compared to the other plasmepsins (e.g. involved in the digestion of haemoglobin inhibitors), inhibitors designed to target these aspartic proteases might be therefore not effective against plasmepsin V. Fortunately, the HIV-1 protease cleaves a similar motif to that of plasmepsin V, emphasising the suggestion that HIV-1 protease-inhibitors could also be potent against the plasmodial protein [64]. Four promising inhibitors, lopinavir, saquinavir, ritonavir and nelfinavir, have already been tested. Unfortunately they are only revealing a moderate inhibitory profile [64, 120] (Table 1). However, in the future by applying medical chemistry these compounds could lead to a complete inhibition of plasmepsin V, and thereby result in the death of the malaria parasite.

Recently, Kulzer et al. identified a unique parasite-encoded HSP70 (*Pf*HSP70-x), which is exported into the host cytosol forming a complex with *Pf*HSP40s [121]. While in other organisms HSP70/40 complexes are important for protein folding and trafficking [122, 123], Kulzer et al. proposed that *Pf*HSP70-x is involved in refolding of other exported proteins and chaperoning these proteins to their final destination. One possible target might be *Pf*EMP1, which (i) colocalises with *Pf*HSP70-x [121] and (ii) is suggested to be transported via a multiprotein complex containing chaperones into the host cell [124]. Therefore *Pf*HSP70-x could serve as a promising novel target to interfere with transportation of the major virulence factor of *P. falciparum.*

## 6. Conclusion

As outlined here, an approach for identifying novel drug targets is represented by proteins which are trafficked within the parasite or its host cell as well as the mechanism of trafficking. For the survival *Plasmodium* is carrying out metabolic processes in intracellular compartments, such as the food vacuole or the apicoplast. Among the targets within the food vacuole, several proteases can be inhibited by different compounds which selectively interfere with the proteolytic digestion of the imported haemoglobin at low micromolar level (Table 1). The apicoplast is an organelle which exhibits metabolic pathways, such as the essential isoprenoid biosynthesis [125]. This pathway has already been exploited as drug target using fosmidomycin (Table 1) [12].

A novel mode to impede proliferation of the malaria pathogen is to prevent proper processing of transport sequences of the trafficked proteins. Plasmepsin V has been found to participate in the posttranslational cleavage of exportation sequences [119], and some promising inhibitors were designed to inhibit plasmepsin V, unfortunately—so far—with only a moderate effect on the parasite's growth. In the future, these molecules might lead to novel inhibitors that prevent host cell modification by *P. falciparum *[64, 120]. However, further discoveries and adaptations of the current drugs are required to hamper proliferation of the deadly human malaria parasite. 

## Figures and Tables

**Table 1:** List of inhibitors known to interfere with protein trafficking or trafficked proteins in *P. falciparum*.

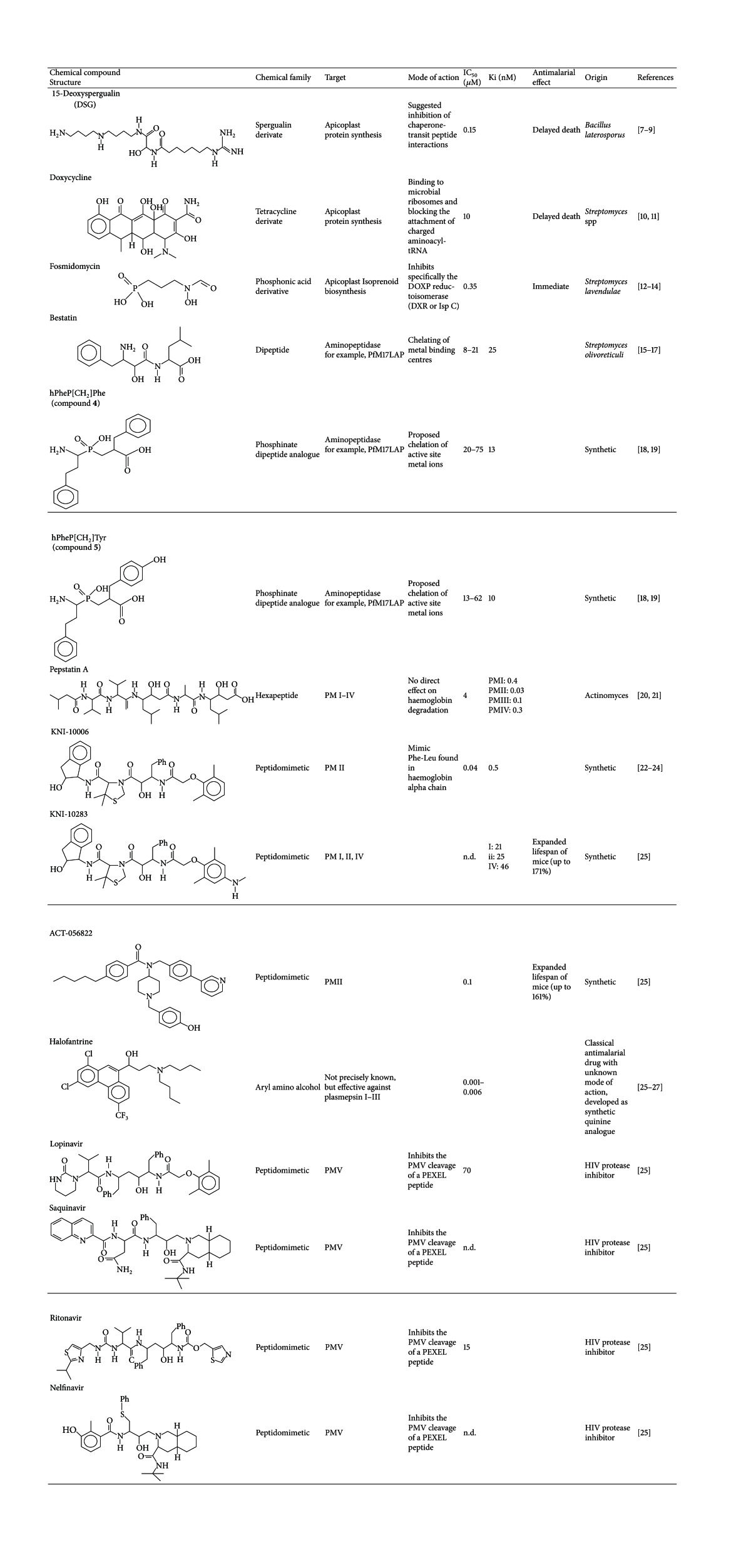

The given IC_50_ values refer to the cellular level. Not yet determined: n.d.

## References

[1] World Health Organization (WHO) (2012). *World Malaria Report 2011*.

[2] Sachs J, Malaney P (2002). The economic and social burden of malaria. *Nature*.

[3] Hecht D, Fogel GB (2012). Modeling the evolution of drug resistance in malaria. *Journal of Computer-Aided Molecular Design*.

[4] Murray CJ, Rosenfeld LC, Lim SS (2012). Global malaria mortality between 1980 and 2010: a systematic analysis. *The Lancet*.

[5] Schumacher RF, Spinelli E (2012). Malaria in children. *Mediterranean Journal of Hematology and Infectious Diseases*.

[6] Nosten F, Rogerson SJ, Beeson JG, McGready R, Mutabingwa TK, Brabin B (2004). Malaria in pregnancy and the endemicity spectrum: what can we learn?. *Trends in Parasitology*.

[7] Ramya TNC, Karmodiya K, Surolia A, Surolia N (2007). 15-deoxyspergualin primarily targets the trafficking of apicoplast proteins in *Plasmodium falciparum*. *Journal of Biological Chemistry*.

[8] Ramya TNC, Surolia N, Surolia A (2006). 15-deoxyspergualin modulates *Plasmodium falciparum* heat shock protein function. *Biochemical and Biophysical Research Communications*.

[9] Banerjee T, Singh RR, Gupta S, Surolia A, Surolia N (2012). 15-deoxyspergualin hinders physical interaction between basic residues of transit peptide in PfENR and Hsp70-1. *IUBMB Life*.

[10] Briolant S, Wurtz N, Zettor A, Rogier C, Pradines B (2010). Susceptibility of *Plasmodium falciparum* isolates to doxycycline is associated with pftetQ sequence polymorphisms and pftetQ and pfmdt copy numbers. *Journal of Infectious Diseases*.

[11] Goodman CD, Su V, McFadden GI (2007). The effects of anti-bacterials on the malaria parasite *Plasmodium falciparum*. *Molecular and Biochemical Parasitology*.

[12] Jomaa H, Wiesner J, Sanderbrand S (1999). Inhibitors of the nonmevalonate pathway of isoprenoid biosynthesis as antimalarial drugs. *Science*.

[13] Botte CY, Dubar F, McFadden GI, Maréchal E, Biot C (2012). *Plasmodium falciparum* apicoplast drugs: targets or off-targets?. *Chemical Reviews*.

[14] Okuhara M, Kuroda Y, Goto T (1980). Studies on new phosphonic acid antibiotics. I. FR-900098, isolation and characterization. *Journal of Antibiotics*.

[15] Gavigan CS, Dalton JP, Bell A (2001). The role of aminopeptidases in haemoglobin degradation in *Plasmodium falciparum*-infected erythrocytes. *Molecular and Biochemical Parasitology*.

[16] Nankya-Kitaka MF, Curley GP, Gavigan CS, Bell A, Dalton JP (1998). Plasmodium chabaudi chabaudi and *P. falciparum*: inhibition of aminopeptidase anti parasite growth by bestatin and nitrobestatin. *Parasitology Research*.

[17] Skinner-Adams TS, Lowther J, Teuscher F (2007). Identification of phosphinate dipeptide analog inhibitors directed against the *Plasmodium falciparum* M17 leucine aminopeptidase as lead antimalarial compounds. *Journal of Medicinal Chemistry*.

[18] McGowan S, Porter CJ, Lowther J (2009). Structural basis for the inhibition of the essential *Plasmodium falciparum* M1 neutral aminopeptidase. *Proceedings of the National Academy of Sciences of the United States of America*.

[19] Grembecka J, Mucha A, Cierpicki T, Kafarski P (2003). The most potent organophosphorus inhibitors of leucine aminopeptidase. Structure-based design, chemistry, and activity. *Journal of Medicinal Chemistry*.

[20] Francis SE, Gluzman LY, Oksman A (1994). Molecular characterization and inhibition of a *Plasmodium falciparum* aspartic hemoglobinase. *EMBO Journal*.

[21] Bailly E, Jambou R, Savel J, Jaureguiberry G (1992). *Plasmodium falciparum*: differential sensitivity *in vitro* to E-64 (cysteine protease inhibitor) and Pepstatin A (aspartyl protease inhibitor). *Journal of Protozoology*.

[22] Hidaka K, Kimura T, Ruben AJ (2008). Antimalarial activity enhancement in hydroxymethylcarbonyl (HMC) isostere-based dipeptidomimetics targeting malarial aspartic protease plasmepsin. *Bioorganic & Medicinal Chemistry*.

[23] Nezami A, Kimura T, Hidaka K (2003). High-affinity inhibition of a family of *Plasmodium falciparum* proteases by a designed adaptive inhibitor. *Biochemistry*.

[24] Miura T, Hidaka K, Uemura T (2010). Improvement of both plasmepsin inhibitory activity and antimalarial activity by 2-aminoethylamino substitution. *Bioorganic and Medicinal Chemistry Letters*.

[25] Meyers MJ, Goldberg DE (2012). Recent advances in plasmepsin medicinal chemistry and implications for future antimalarial drug discovery efforts. *Current Topics in Medicinal Chemistry*.

[26] Ndong JMM, Atteke C, Aubouy A, Bakary M, Lébibi J, Deloron P (2003). *In vitro* activity of chloroquine, quinine, mefloquine and halofantrine against Gabonese isolates of *Plasmodium falciparum*. *Tropical Medicine and International Health*.

[27] Sidhu ABS, Uhlemann AC, Valderramos SG, Valderramos JC, Krishna S, Fidock DA (2006). Decreasing pfmdr1 copy number in *Plasmodium falciparum* malaria heightens susceptibility to mefloquine, lumefantrine, halofantrine, quinine, and artemisinin. *Journal of Infectious Diseases*.

[28] Uhlemann AC, Krishna S (2005). Antimalarial multi-drug resistance in Asia: mechanisms and assessment. *Current Topics in Microbiology and Immunology*.

[29] Mita T, Tanabe K (2012). Evolution of *Plasmodium falciparum* drug resistance: implications for the development and containment of artemisinin resistance. *Japanese Journal of Infectious Diseases*.

[30] Mugittu K, Ndejembi M, Malisa A (2004). Therapeutic efficacy of sulfadoxine-pyrimethamine and prevalence of resistance markers in Tanzania prior to revision of malaria treatment policy: *Plasmodium falciparum* dihydrofolate reductase and dihydropteroate synthase mutations in monitoring *in vivo* resistance. *American Journal of Tropical Medicine and Hygiene*.

[31] Marks F, von Kalckreuth V, Kobbe R (2005). Parasitological rebound effect and emergence of pyrimethamine resistance in *Plasmodium falciparum* after single-dose sulfadoxine-pyrimethamine. *Journal of Infectious Diseases*.

[32] Fivelman QL, Butcher GA, Adagu IS, Warhurst DC, Pasvol G (2002). Malarone treatment failure and in vitro confirmation of resistance of *Plasmodium falciparum* isolate from Lagos, Nigeria. *Malaria Journal*.

[33] White NJ (2008). Qinghaosu (artemisinin): the price of success. *Science*.

[34] Witkowski B, Khim N, Chim P Reduced artemisinin susceptibility of *Plasmodium falciparum* ring stages in western Cambodia.

[35] Ch'ng JH, Liew K, Goh AS, Sidhartha E, Tan KS (2011). Drug-induced permeabilization of parasite's digestive vacuole is a key trigger of programmed cell death in *Plasmodium falciparum*. *Cell Death & Disease*.

[36] Dondorp AM, Nosten F, Yi P (2009). Artemisinin resistance in *Plasmodium falciparum* malaria. *The New England Journal of Medicine*.

[37] Rosenthal PJ, McKerrow JH, Aikawa M, Nagasawa H, Leech JH (1988). A malarial cysteine proteinase is necessary for hemoglobin degradation by *Plasmodium falciparum*. *Journal of Clinical Investigation*.

[38] Maier AG, Rug M, O’Neill MT (2008). Exported proteins required for virulence and rigidity of *Plasmodium falciparum*-infected human erythrocytes. *Cell*.

[39] Hanssen E, McMillan PJ, Tilley L (2010). Cellular architecture of *Plasmodium falciparum*-infected erythrocytes. *International Journal for Parasitology*.

[40] Köhler S, Delwiche CF, Denny PW (1997). A plastid of probable green algal origin in Apicomplexan parasites. *Science*.

[41] Wilson RJMI, Denny PW, Preiser PR (1996). Complete gene map of the plastid-like DNA of the malaria parasite *Plasmodium falciparum*. *Journal of Molecular Biology*.

[42] Foth BJ, Ralph SA, Tonkin CJ (2003). Dissecting apicoplast targeting in the malaria parasite *Plasmodium falciparum*. *Science*.

[43] Carlton JM, Angiuoli SV, Suh BB (2002). Genome sequence and comparative analysis of the model rodent malaria parasite *Plasmodium yoelii yoelii*. *Nature*.

[44] Waller RF, Keeling PJ, Donald RGK (1998). Nuclear-encoded proteins target to the plastid in *Toxoplasma gondii* and *Plasmodium falciparum*. *Proceedings of the National Academy of Sciences of the United States of America*.

[45] Blobel G (1980). Intracellular protein topogenesis. *Proceedings of the National Academy of Sciences of the United States of America*.

[46] von Heijne G (1984). How signal sequences maintain cleavage specificity. *Journal of Molecular Biology*.

[47] Spiess M (1995). Heads or tails—what determines the orientation of proteins in the membrane. *FEBS Letters*.

[48] Bannykh SI, Rowe T, Balch WE (1996). The organization of endoplasmic reticulum export complexes. *Journal of Cell Biology*.

[49] Waller RF, Reed MB, Cowman AF, McFadden GI (2000). Protein trafficking to the plastid of *Plasmodium falciparum* is via the secretory pathway. *EMBO Journal*.

[50] Ehlgen F, Pham JS, de Koning-Ward T, Cowman AF, Ralph SA (2012). Investigation of the *Plasmodium falciparum* food vacuole through inducible expression of the chloroquine resistance transporter (PfCRT). *PLoS One*.

[51] Albano FR, Berman A, Greca La N (1999). A homologue of Sar1p localises to a novel trafficking pathway in malaria-infected erythrocytes. *European Journal of Cell Biology*.

[52] Adisa A, Albano FR, Reeder J, Foley M, Tilley L (2001). Evidence for a role for a *Plasmodium falciparum* homologue of Sec31p in the export of proteins to the surface of malaria parasite-infected erythrocytes. *Journal of Cell Science*.

[53] Adisa A, Rug M, Klonis N, Foley M, Cowman AF, Tilley L (2003). The signal sequence of exported protein-1 directs the green fluorescent protein to the parasitophorous vacuole of transfected malaria parasites. *Journal of Biological Chemistry*.

[54] Müller IB, Knöckel J, Eschbach ML, Bergmann B, Walter RD, Wrenger C (2010). Secretion of an acid phosphatase provides a possible mechanism to acquire host nutrients by *Plasmodium falciparum*. *Cellular Microbiology*.

[55] Klemba M, Gluzman I, Goldberg DE (2004). A *Plasmodium falciparum* dipeptidyl aminopeptidase I participates in vacuolar hemoglobin degradation. *Journal of Biological Chemistry*.

[56] Francis SE, Banerjee R, Goldberg DE (1997). Biosynthesis and maturation of the malaria aspartic hemoglobinases plasmepsins I and II. *Journal of Biological Chemistry*.

[57] Banerjee R, Francis SE, Goldberg DE (2003). Food vacuole plasmepsins are processed at a conserved site by an acidic convertase activity in *Plasmodium falciparum*. *Molecular and Biochemical Parasitology*.

[58] McIntosh MT, Vaid A, Hosgood HD (2007). Traffic to the malaria parasite food vacuole: a novel pathway involving a phosphatidylinositol 3-phosphate-binding protein. *Journal of Biological Chemistry*.

[59] Dasaradhi PVN, Korde R, Thompson JK (2007). Food vacuole targeting and trafficking of falcipain-2, an important cysteine protease of human malaria parasite *Plasmodium falciparum*. *Molecular and Biochemical Parasitology*.

[60] Subramanian S, Sijwali PS, Rosenthal PJ (2007). Falcipain cysteine proteases require bipartite motifs for trafficking to the *Plasmodium falciparum* food vacuole. *Journal of Biological Chemistry*.

[61] Hiller NL, Bhattacharjee S, van Ooij C (2004). A host-targeting signal in virulence proteins reveals a secretome in malarial infection. *Science*.

[62] Marti M, Good RT, Rug M, Knuepfer E, Cowman AF (2004). Targeting malaria virulence and remodeling proteins to the host erythrocyte. *Science*.

[63] Sargeant TJ, Marti M, Caler E (2006). Lineage-specific expansion of proteins exported to erythrocytes in malaria parasites. *Genome Biology*.

[64] Boddey JA, Hodder AN, Günther S (2010). An aspartyl protease directs malaria effector proteins to the host cell. *Nature*.

[65] de Koning-Ward TF, Gilson PR, Boddey JA (2009). A newly discovered protein export machine in malaria parasites. *Nature*.

[66] Bullen HE, Charnaud SC, Kalanon M (2012). Biosynthesis, localization, and macromolecular arrangement of the *Plasmodium falciparum* translocon of exported proteins (PTEX). *The Journal of Biological Chemistry*.

[67] Spielmann T, Gilberger TW (2010). Protein export in malaria parasites: do multiple export motifs add up to multiple export pathways?. *Trends in Parasitology*.

[68] Waller RF, McFadden GI (2005). The apicoplast: a review of the derived plastid of apicomplexan parasites. *Current Issues in Molecular Biology*.

[69] Seeber F (2002). Biogenesis of iron-sulphur clusters in amitochondriate and apicomplexan protists. *International Journal for Parasitology*.

[70] Seeber F (2003). Biosynthetic pathways of plastid-derived organelles as potential drug targets against parasitic apicomplexa. *Current Drug Targets. Immune, Endocrine and Metabolic Disorders*.

[71] Kumar B, Chaubey S, Shah P (2011). Interaction between sulphur mobilisation proteins SufB and SufC: evidence for an iron-sulphur cluster biogenesis pathway in the apicoplast of *Plasmodium falciparum*. *International Journal for Parasitology*.

[72] van Dooren GG, Schwartzbach SD, Osafune T, McFadden GI (2001). Translocation of proteins across the multiple membranes of complex plastids. *Biochimica et Biophysica Acta—Molecular Cell Research*.

[73] Heard TS, Weiner H (1998). A regional net charge and structural compensation model to explain how negatively charged amino acids can be accepted within a mitochondrial leader sequence. *Journal of Biological Chemistry*.

[74] Ni L, Heard TS, Weiner H (1999). *In vivo* mitochondrial import: a comparison of leader sequence charge and structural relationships with the *in vitro* model resulting in evidence for co-translational import. *Journal of Biological Chemistry*.

[75] Ralph SA, Foth BJ, Hall N, McFadden GI (2004). Evolutionary pressures on apicoplast transit peptides. *Molecular Biology and Evolution*.

[76] Tonkin CJ, Roos DS, McFadden GI (2006). N-terminal positively charged amino acids, but not their exact position, are important for apicoplast transit peptide fidelity in Toxoplasma gondii. *Molecular and Biochemical Parasitology*.

[77] Kalanon M, Tonkin CJ, McFadden GI (2009). Characterization of two putative protein translocation components in the apicoplast of *Plasmodium falciparum*. *Eukaryotic Cell*.

[78] Gillece P, Luz JM, Lennarz WJ, de La Cruz FJ, Römisch K (1999). Export of a cysteine-free misfolded secretory protein from the endoplasmic reticulum for degradation requires interaction with protein disulfide isomerase. *Journal of Cell Biology*.

[79] Wahlman J, DeMartino GN, Skach WR, Bulleid NJ, Brodsky JL, Johnson A (2007). Real-time fluorescence detection of ERAD substrate retrotranslocation in a mammalian *in vitro* system. *Cell*.

[80] Tsai B, Rodighiero C, Lencer WI, Rapoport TA (2001). Protein disulfide isomerase acts as a redox-dependent chaperone to unfold cholera toxin. *Cell*.

[81] Wiertz EJHJ, Tortorella D, Bogyo M (1996). Sec61-mediated transfer of a membrane protein from the endoplasmic reticulum to the proteasome for destruction. *Nature*.

[82] Knop M, Finger A, Braun T, Hellmuth K, Wolf DH (1996). Der1, a novel protein specifically required for endoplasmic reticulum degradation in yeast. *EMBO Journal*.

[83] Borrmann S, Issifou S, Esser G (2004). Fosmidomycin-clindamycin for the treatment of *Plasmodium falciparum* malaria. *Journal of Infectious Diseases*.

[84] Ivey RA, Bruce BD (2000). *In vivo* and *in vitro* interaction of DnaK and a chloroplast transit peptide. *Cell Stress and Chaperones*.

[85] Ivey RA, Subramanian C, Bruce BD (2000). Identification of a Hsp70 recognition domain within the Rubisco small subunit transit peptide. *Plant Physiology*.

[86] Wiesner J, Reichenberg A, Heinrich S, Schlitzer M, Jomaa H (2008). The plastid-like organelle of apicomplexan parasites as drug target. *Current Pharmaceutical Design*.

[87] Sousa M, Pozniak A, Boffito M (2008). Pharmacokinetics and pharmacodynamics of drug interactions involving rifampicin, rifabutin and antimalarial drugs. *Journal of Antimicrobial Chemotherapy*.

[88] Ralph SA, van Dooren GG, Waller RF (2004). Tropical infectious diseases: metabolic maps and functions of the *Plasmodium falciparum* apicoplast. *Nature Reviews Microbiology*.

[89] Couto AS, Kimura EA, Peres VJ, Uhrig ML, Katzin AM (1999). Active isoprenoid pathway in the intra-erythrocytic stages of *Plasmodium falciparum*: presence of dolichols of 11 and 12 isoprene units. *Biochemical Journal*.

[90] Geist JG, Lauw S, Illarionova V (2010). Thiazolopyrimidine inhibitors of 2-methylerythritol 2,4-cyclodiphosphate synthase (IspF) from *Mycobacterium tuberculosis* and *Plasmodium falciparum*. *ChemMedChem*.

[91] He CY, Striepen B, Pletcher CH, Murray JM, Roos DS (2001). Targeting and processing of nuclear-encoded apicoplast proteins in plastid segregation mutants of *Toxoplasma gondii*. *Journal of Biological Chemistry*.

[92] Kobayashi T, Sato S, Takamiya S (2007). Mitochondria and apicoplast of *Plasmodium falciparum*: behaviour on subcellular fractionation and the implication. *Mitochondrion*.

[93] Moe MK, Samuelsen PJ, Nielsen HV, Nielsen KM (2010). Separation of DNA-containing organelles from *Toxoplasma gondii* by CZE. *Electrophoresis*.

[94] Odom AR, van Voorhis WC (2010). Functional genetic analysis of the *Plasmodium falciparum* deoxyxylulose 5-phosphate reductoisomerase gene. *Molecular and Biochemical Parasitology*.

[95] Dalal S, Klemba M (2007). Roles for two aminopeptidases in vacuolar hemoglobin catabolism in *Plasmodium falciparum*. *Journal of Biological Chemistry*.

[96] Ragheb D, Bompiani K, Dalal S, Klemba M (2009). Evidence for catalytic roles for *Plasmodium falciparum* aminopeptidase P in the food vacuole and cytosol. *Journal of Biological Chemistry*.

[97] Ragheb D, Dalal S, Bompiani KM, Ray WK, Klemba M (2011). Distribution and biochemical properties of an M1-family aminopeptidase in *Plasmodium falciparum* indicate a role in vacuolar hemoglobin catabolism. *Journal of Biological Chemistry*.

[98] Gluzman IY, Francis SE, Oksman A, Smith CE, Duffin KL, Goldberg DE (1994). Order and specificity of the *Plasmodium falciparum* hemoglobin degradation pathway. *Journal of Clinical Investigation*.

[99] Banerjee R, Liu J, Beatty W, Pelosof L, Klemba M, Goldberg DE (2002). Four plasmepsins are active in the *Plasmodium falciparum* food vacuole, including a protease with an active-site histidine. *Proceedings of the National Academy of Sciences of the United States of America*.

[100] Wyatt DM, Berry C (2002). Activity and inhibition of plasmepsin IV, a new aspartic proteinase from the malaria parasite, *Plasmodium falciparum*. *FEBS Letters*.

[101] Shenai BR, Sijwali PS, Singh A, Rosenthal PJ (2000). Characterization of native and recombinant falcipain-2, a principal trophozoite cysteine protease and essential hemoglobinase of *Plasmodium falciparum*. *Journal of Biological Chemistry*.

[102] Sijwali PS, Brinen LS, Rosenthal PJ (2001). Systematic optimization of expression and refolding of the *Plasmodium falciparum* cysteine protease falcipain-2. *Protein Expression and Purification*.

[103] Eggleson KK, Duffin KL, Goldberg DE (1999). Identification and characterization of falcilysin, a metallopeptidase involved in hemoglobin catabolism within the malaria parasite *Plasmodium falciparum*. *Journal of Biological Chemistry*.

[104] Kolakovich KA, Gluzman IY, Duffin KL, Goldberg DE (1997). Generation of hemoglobin peptides in the acidic digestive vacuole of *Plasmodium falciparum* implicates peptide transport in amino acid production. *Molecular and Biochemical Parasitology*.

[105] Shea M, Jäkle U, Liu Q, Berry C, Joiner KA, Soldati-Favre D (2007). A family of aspartic proteases and a novel, dynamic and cell-cycle-dependent protease localization in the secretory pathway of *Toxoplasma gondii*. *Traffic*.

[106] Skinner-Adams TS, Stack CM, Trenholme KR (2010). *Plasmodium falciparum* neutral aminopeptidases: new targets for anti-malarials. *Trends in Biochemical Sciences*.

[107] Ziegler J, Linck R, Wright DW (2001). Heme aggregation inhibitors: antimalarial drugs targeting an essential biomineralization process. *Current Medicinal Chemistry*.

[108] Liu J, Gluzman IY, Drew ME, Goldberg DE (2005). The role of *Plasmodium falciparum* food vacuole plasmepsins. *Journal of Biological Chemistry*.

[109] Bonilla JA, Bonilla TD, Yowell CA, Fujioka H, Dame JB (2007). Critical roles for the digestive vacuole plasmepsins of *Plasmodium falciparum* in vacuolar function. *Molecular Microbiology*.

[110] Moura PA, Dame JB, Fidock DA (2009). Role of *Plasmodium falciparum* digestive vacuole plasmepsins in the specificity and antimalarial mode of action of cysteine and aspartic protease inhibitors. *Antimicrobial Agents and Chemotherapy*.

[111] Ruben AJK, Y AJK, Freire E, Ghosh AKE (2010). The plasmepsin family as antimalarial drug targets. *Aspartic Acid Proteases as Therapeutic Targets*.

[112] Gil LA, Valiente PA, Pascutti PG, Pons T (2011). Computational perspectives into plasmepsins structure-function relationship: implications to inhibitors design. *Journal of Tropical Medicine*.

[113] Ahmed W, Rani M, Khan IA (2010). Characterisation of hydrazides and hydrazine derivatives as novel aspartic protease inhibitors. *Journal of Enzyme Inhibition and Medicinal Chemistry*.

[114] McKay PB, Peters MB, Carta G (2011). Identification of plasmepsin inhibitors as selective anti-malarial agents using ligand based drug design. *Bioorganic and Medicinal Chemistry Letters*.

[115] Kasam V, Zimmermann M, Maaß A (2007). Design of new plasmepsin inhibitors: a virtual high throughput screening approach on the EGEE grid. *Journal of Chemical Information and Modeling*.

[116] Degliesposti G, Kasam V, da Costa A (2009). Design and discovery of plasmepsin II inhibitors using an automated workflow on large-scale grids. *ChemMedChem*.

[117] Friedman R, Caflisch A (2009). Discovery of plasmepsin inhibitors by fragment-based docking and consensus scoring. *ChemMedChem*.

[118] Su XZ, Heatwole VM, Wertheimer SP (1995). The large diverse gene family var encodes proteins involved in cytoadherence and antigenic variation of *Plasmodium falciparum*-infected erythrocytes. *Cell*.

[119] Guruprasad L, Tanneeru K, Guruprasad K (2011). Structural rationale for the Recognition of Arginine at P3 in PEXEL motif containing proteins of *Plasmodium falciparum* by plasmepsin V. *Protein and Peptide Letters*.

[120] Russo I, Babbitt S, Muralidharan V, Butler T, Oksman A, Goldberg DE (2010). Plasmepsin V licenses Plasmodium proteins for export into the host erythrocyte. *Nature*.

[121] Kulzer S, Charnaud S, Dagan T (2012). *Plasmodium falciparum*-encoded exported hsp70/hsp40 chaperone/co-chaperone complexes within the host erythrocyte. *Cellular Microbiology*.

[122] Kampinga HH, Craig EA (2010). The HSP70 chaperone machinery: J proteins as drivers of functional specificity. *Nature Reviews Molecular Cell Biology*.

[123] Hegde RS, Keenan RJ (2011). Tail-anchored membrane protein insertion into the endoplasmic reticulum. *Nature Reviews Molecular Cell Biology*.

[124] Knuepfer E, Rug M, Klonis N, Tilley L, Cowman AF (2005). Trafficking of the major virulence factor to the surface of transfected *P falciparum*-infected erythrocytes. *Blood*.

[125] Yeh E, DeRisi JL (2011). Chemical rescue of malaria parasites lacking an apicoplast defines organelle function in blood-stage *Plasmodium falciparum*. *PLoS Biology*.

